# A neural marker of rapid discrimination of facial expression in 3.5- and 7-month-old infants

**DOI:** 10.3389/fnins.2022.901013

**Published:** 2022-08-18

**Authors:** Fanny Poncet, Arnaud Leleu, Diane Rekow, Fabrice Damon, Milena P. Dzhelyova, Benoist Schaal, Karine Durand, Laurence Faivre, Bruno Rossion, Jean-Yves Baudouin

**Affiliations:** ^1^Development of Olfactory Communication and Cognition Laboratory, Centre des Sciences du Goût et de l’Alimentation, CNRS, Université Bourgogne Franche-Comté, INRAE, Institut Agro, Dijon, France; ^2^Université Grenoble Alpes, Saint-Martin-d’Hères, France; ^3^Université de Lorraine, CNRS, CRAN–UMR 7039, Nancy, France; ^4^Inserm UMR 1231 GAD, Genetics of Developmental Disorders, and Centre de Référence Maladies Rares “Anomalies du Développement et Syndromes Malformatifs,” FHU TRANSLAD, CHU Dijon and Université de Bourgogne-Franche Comté, Dijon, France; ^5^Service de Neurologie, Université de Lorraine, CHRU-Nancy, Nancy, France; ^6^Laboratoire “Développement, Individu, Processus, Handicap, Éducation”, Département Psychologie du Développement, de l’Éducation et des Vulnérabilités, Institut de Psychologie, Université de Lyon, Université Lumière Lyon 2, Bron, France

**Keywords:** infant, visual perception, facial expression of emotions, fast periodic visual stimulation, EEG, development

## Abstract

Infants’ ability to discriminate facial expressions has been widely explored, but little is known about the rapid and automatic ability to discriminate a given expression against many others in a single experiment. Here we investigated the development of facial expression discrimination in infancy with fast periodic visual stimulation coupled with scalp electroencephalography (EEG). EEG was recorded in eighteen 3.5- and eighteen 7-month-old infants presented with a female face expressing disgust, happiness, or a neutral emotion (in different stimulation sequences) at a base stimulation frequency of 6 Hz. Pictures of the same individual expressing other emotions (either anger, disgust, fear, happiness, sadness, or neutrality, randomly and excluding the expression presented at the base frequency) were introduced every six stimuli (at 1 Hz). Frequency-domain analysis revealed an objective (i.e., at the predefined 1-Hz frequency and harmonics) expression-change brain response in both 3.5- and 7-month-olds, indicating the visual discrimination of various expressions from disgust, happiness and neutrality from these early ages. At 3.5 months, the responses to the discrimination from disgust and happiness expressions were located mainly on medial occipital sites, whereas a more lateral topography was found for the response to the discrimination from neutrality, suggesting that expression discrimination from an emotionally neutral face relies on distinct visual cues than discrimination from a disgust or happy face. Finally, expression discrimination from happiness was associated with a reduced activity over posterior areas and an additional response over central frontal scalp regions at 7 months as compared to 3.5 months. This result suggests developmental changes in the processing of happiness expressions as compared to negative/neutral ones within this age range.

## Introduction

A large body of research suggests that the first year of life is critical in the development of facial emotion perception [e.g., Campos et al. (1983); for reviews, see Nelson (1987); Leppänen and Nelson (2009), and Maria et al. (2018)]. Some studies have reported early abilities for discriminating facial expressions (e.g., Field et al., 1982; Farroni et al., 2007; Addabbo et al., 2018), while other studies have challenged this view [Kaitz et al., 1988; Oostenbroek et al., 2016; see also Soussignan et al. (2018)]. In fact, the ability of infants to discriminate, recognize, and adapt their own behavior to the facial expressions of others seems to develop gradually over infancy and childhood. For most facial expressions, while discriminative abilities have been evidenced at an early age, the recognition of a specific emotion and its meaning has not been clearly established before the second half of the first year (Walker-Andrews, 1997; Leppänen and Nelson, 2009).

Many authors have delineated significant changes in the processing of emotional facial expression during the first year (Campos and Stenberg, 1981; Oster, 1981; Walker-Andrews, 1997, 2005; Leppänen and Nelson, 2009; Leppänen, 2011; Quinn et al., 2011). Infants seem to start differentiating facial expressions by progressively decoding specific cues and configurations, before being able to attribute emotional meaning to specific patterns of facial actions contingent with repeated social interactions. For instance, some studies reveal that newborns discriminate between dynamic facial expressions of happy and disgusted [but only after being habituated to a happy or disgusted one: Addabbo et al. (2018)]. From 3 to 4 months of age, infants visually discriminate some facial expressions, especially happiness from frowning, anger, sadness, or neutrality (LaBarbera et al., 1976; Young-Browne et al., 1977; Barrera and Maurer, 1981; Haviland and Lelwica, 1987). However, they do not reliably “categorize” facial expressions (i.e., generalize an emotion category across different identities or views) before 5–7 months of age (Caron et al., 1982; Serrano et al., 1992; Kotsoni et al., 2001; Bornstein and Arterberry, 2003; Bornstein et al., 2011). Infants react to positive emotions (smiling more to happy than to neutral and negative faces) from about 3 months of age (e.g., Millar, 1976; Legerstee, 1997; Soussignan et al., 2018), and progressively extend this reaction to negative emotions during the second part of the first year [e.g., Kaiser et al. (2017); for reviews, see Campos and Stenberg (1981) and Oster (1981)]. In particular, with the development of referential looking behaviors between 7 and 12 months of age (Rochat and Striano, 1999), infants progressively refer to the negative facial expressions of adults and adjust their behavior accordingly (Feinman and Lewis, 1983; Klinnert et al., 1986; Campos et al., 2003). For instance, fearful faces elicit an adult-like attentional orienting over neutral or happy faces in 7-month-olds (e.g., Nelson et al., 1979; Nelson and Dolgin, 1985; Kotsoni et al., 2001; Peltola et al., 2008, 2009, 2013). It is generally suggested that the ability to assign meaning to facial expressions emerges from this age of 7 months onward, after experience-expectant developmental processes (Leppänen and Nelson, 2009; Leppänen, 2011).

In line with the behavioral literature (Walker-Andrews, 1997; Quinn et al., 2011), studies on brain activity further indicate critical differences between the different expressions in the early development of facial expression processing from the middle of the first year. At 7 months of age, an “adult-like neural circuitry” is engaged to process some emotional faces [sad and happy faces: Rotem-Kohavi et al. (2017); fearful faces: Leppänen and Nelson (2009)]. EEG studies have revealed that, as in adults, looking facial expressions elicits sensorimotor activity at 7 months of age, but only for happy faces (Quadrelli et al., 2021). At this age, the brain response to happiness is different from negative expressions like angry faces for the temporal aspect (both right-lateralized), and the response to angry faces is associated with higher scores on a Negative Affect temperamental dimension (Quadrelli et al., 2019). In both these two studies, the stimulus dynamicity has been observed as more efficiently processed by functional brain networks at this age as compared to static stimuli. For fear, modulations were observed over medial occipital and occipito-temporal sites (i.e., related to the visual processing of faces) in studies measuring event-related potentials (ERPs) (e.g., de Haan and Nelson, 1999; Halit et al., 2004; Leppänen et al., 2007; Xie et al., 2019). Other modulations were reported over central frontal regions in response to fear, happiness or anger in ERP studies [Nelson and de Haan (1996); Leppänen et al. (2007), and Xie et al. (2019): with an effect emerging at 5 months of age before becoming well-established at 7 months of age] or studies recording functional near-infrared spectroscopy [fNIRS; Minagawa-Kawai et al. (2008); Fox et al. (2013), and Bayet et al. (2021): especially for happiness over temporo-parietal sites]. ERP studies relate attentional orienting toward salient stimuli (Nelson, 1994; de Haan, 2007) to cortical sources either in the prefrontal and anterior cingulate cortices (Reynolds and Richards, 2005), or in the posterior cingulate cortex/precuneus and temporal areas (Guy et al., 2016; Xie et al., 2019). In fNIRS studies, greater activity occurs in the medial prefrontal cortex (mPFC) for smiling over neutral faces [in 7-month-olds: Fox et al. (2013); in 9- to 13-month-olds: Minagawa-Kawai et al. (2008)], the mPFC being credited to play an important role in the early acquisition of socio-cognitive skills (Grossmann, 2013).

Overall, the studies reviewed above suggest that the nature and topography of the brain response to facial expressions differ between the facial expressions, at least in infants in the second half of the first year [that could start to emerge at 5 months: e.g., Xie et al. (2019)], with posterior responses, temporo-parietal and/or central frontal regions according to the emotional expressions. Some studies suggest that more central frontal responses emerge with age, posterior regions responding more at an earlier stage (e.g., Xie et al., 2019). However, studies using standard ERP and fNIRS approaches report quite variable results, with the difficulty of isolating clear brain responses to the discrimination of different facial expressions, as also noted in adult studies [for reviews on the ERP approach on this topic, see Vuilleumier and Pourtois (2007) and Calvo and Nummenmaa (2016)]. To overcome this limitation, recent studies in adults have used fast periodic visual stimulation (FPVS) coupled with scalp EEG. Robust and specific neural responses to brief expression changes were isolated, each emotional expression being directly contrasted to a neutral face (e.g., Dzhelyova et al., 2017; Leleu et al., 2018; Matt et al., 2021) or to all other expressions (Poncet et al., 2019). This FPVS-EEG approach relies on the property of the brain to synchronize with stimuli displayed periodically (Adrian and Matthews, 1934), eliciting EEG responses at the same frequency [Regan (1989) and Norcia et al. (2015), for reviews]. This allows isolating an objective response (i.e., measured at a predefined frequency of stimulation) to a specific visual content in a few minutes of recording. By presenting stimuli at a rapid rate (i.e., the base frequency) and introducing a specific type of target stimuli periodically at a slower rate, a variation of this approach isolates a brain response that directly reflects the difference between the target stimuli and the base stimuli (i.e., without *post hoc* subtraction) [for review see Rossion et al. (2020)]. While this approach has been used to isolate face categorization abilities in the infant brain [de Heering and Rossion, 2015; Peykarjou et al., 2017; Leleu et al., 2020; Rekow et al., 2020, 2021; see also Barry-Anwar et al. (2018)], to date, it has not been used to measure the discrimination of facial expressions of emotion in this population.

**FIGURE 1:**
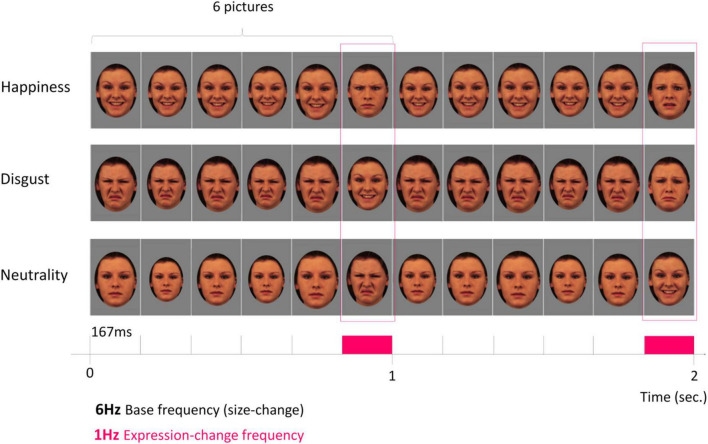
Fast periodic visual stimulation in electroencephalography (FPVS-EEG) to isolate a neural marker of rapid discrimination of facial expression. Among the five basic emotional facial expressions used (disgust, happiness, anger, fear, sadness) and neutrality, the discrimination of three target expressions was tested (disgust, happiness and neutrality). A 2-s sequence of fast periodic stimulation is depicted with images from one individual face. From top to bottom: happy, disgust, and neutral facial expressions are respectively used in dedicated sequences and periodically displayed at the 6-Hz base rate (i.e., six pictures per second; 1 cycle ≈ 167 ms) without inter-stimulus interval, and the five other expressions are randomly displayed at the 1-Hz expression-change frequency (every 6th cycle = 1 s between each expression-change). Images were presented through ±15% randomized size variation at each cycle. This design thus isolates two dissociated responses at two different frequencies: a general visual response (6 Hz) to the rapid train of one individual face varying in size and an expression-change response (1 Hz) reflecting rapid (i.e., single-glance) discrimination of all inserted expressions from the target expression presented at the base rate. Source: KDEF database (models coded 07F, 09F, 14F, and 20F) (Lundqvist et al., 1998).

Here, we used FPVS-EEG to isolate neural responses to the discrimination of specific facial expressions in 3.5- and 7-month-old infants. In particular, our goal was to dissociate the response from one expression to several other expressions in a single stimulation sequence. In classical behavioral or ERP studies, constraints related to the limited attentional availability of infants, combined with the need to have a sufficient number of trials per experimental condition, usually makes it necessary to limit the number of contrasted expressions to avoid an exponential increase in stimulation time or number of participants. With FPVS, it is possible to present one expression at one frequency and all the others at another frequency, without weighting down the procedure. Above all, the dissociation then carried out isolates the *specific* response to the expression, i.e., what differentiates it from *all the others*. For example, in the study by Poncet et al. (2019) on adult participants, every expression was displayed at a base frequency of 6 Hz (i.e., six stimuli per second), and a target expression was interspersed every 6th stimulus (i.e., at a specific frequency of 6/6 = 1 Hz). As a result, the specific brain response to the target expression was dissociated from all the other categories at the 1-Hz frequency. Another procedure to capture the differential response between an information of interest and a baseline control information is to display the first information at the base frequency and the baseline information at the oddball frequency. For example, identity discrimination was evidenced by displaying one identity at the base frequency and multiple other identities at the oddball frequency (e.g., Rossion et al., 2020). Here, we adapted this procedure to infants: the target expression was repeatedly displayed at a base frequency of 6 Hz, and other emotion categories were interspersed every 6th stimulus (at 1 Hz). We opted to present the target expression at the base frequency to reduce the visual variability of the stimulation; within six images, infants were exposed to two distinct expressions (five times the target expression and one time another randomly selected expression). This procedure provides additional time to process the target expression and reduces backward and forward masking effects (Figure 1). Hence, given that the brain response recorded at 1 Hz reflects a generalized *differential* activity elicited by all expression changes within a sequence, it remains a clear marker that the infant brain discriminates the facial expressions inserted at 1 Hz from the target expression displayed at 6 Hz while making the rapid stimulation less challenging for infants. Three facial expressions - neutrality, happiness and disgust–were considered. According to previous studies (e.g., de Haan and Nelson, 1999; Halit et al., 2004; Leppänen et al., 2007; Xie et al., 2019), we first hypothesized that each expression elicits a specific brain response over posterior regions at both ages, reflecting the ability of the infant brain to detect the specific visual characteristics of an expression that differentiates it from other expressions. In addition, according to the progressive integration of affective and socio-cognitive processes in the perception of emotional expressions from the second half of the first year, we expected an evolution of the brain response to the expression that acquired significance between 3.5 and 7 months, i.e., happiness, with the emergence of central frontal responses (Nelson and de Haan, 1996; Leppänen et al., 2007; Minagawa-Kawai et al., 2008; Fox et al., 2013; Xie et al., 2019). By contrast, we used disgust as a “control” expression, since the age at which infants start to understand the meaning of disgust—or whether this ability appears in infancy—is not established during the first year [see Widen and Russell (2010, 2013) and Ruba et al. (2019); even if a discriminative ability is observed in newborns between dynamic faces of disgust and happiness: Addabbo et al. (2018)]. In the literature investigating dynamic presentation of expressions as compared to static ones, evidence have been revealed that although infants from 6-months showed clear diagnostic scanning of expressions (e.g., exploration of lower part of the face, nose and mouth), a developing sophistication in scanning for negative expressions of angry and fearful but also disgusted expressions was observed from 6 to 12 months: Prunty et al. (2021). Therefore, we did not expect an evolution of the response to an expression change from disgust between 3.5 and 7 months.

## Materials and methods

### Participants

Twenty-one 3.5-month-old and twenty-two 7-month-old infants participated in the study. They were recruited by mail through the local birth registry. Before testing, all parents gave written informed consent. Testing was conducted according to the Declaration of Helsinki and approved by a French ethics committee (Comité de Protection des Personnes Sud-Est III–2016-A02056-45). Data from three 3.5- and four 7-month-old infants were excluded from the analyses due to less than two valid sequences for one condition or unusable EEG data because of too noisy signals. The final sample thus consisted of eighteen 3.5-month-olds (four females, mean age ± *SD* = 107.8 ± 4.7 days, range: 101–116 days), and eighteen 7-month-olds (7 females, mean age ± *SD* = 216.8 ± 6.7 days, range: 204–229 days).

### Visual stimuli

Face pictures of four females depicting basic emotional expressions (disgust, happiness, anger, fear, sadness) and neutrality were selected from the KDEF database (models coded 07F, 09F, 14F, and 20F) (Lundqvist et al., 1998). They were equalized in terms of luminance and put into a medallion-shaped window to discard information from the background and hairstyle (Figure 1). They were displayed on a mid-level gray background (i.e., 128/255 in grayscale) with face-size adjusted to 22.6 × 17 cm. Stimuli were presented at the center of a screen at an approximate viewing distance of 57 cm, subtending a large part of the visual field of infants (i.e., 21.6 × 16.6° of visual angle). Hence, the size of the face images was close to the size of faces encountered by infants during typical social interactions (de Heering and Rossion, 2015; Leleu et al., 2020).

### Procedure

The procedure was largely borrowed from experiments that successfully isolated and quantified brain signatures of facial expression categorization in adults using FPVS (Dzhelyova et al., 2017; Leleu et al., 2018; Poncet et al., 2019). Stimuli were presented without inter-stimulus interval on a 24-inch LED screen (60 Hz refresh rate, resolution: 1920 × 1,080 pixels) on a mid-level gray background (i.e., 128/255 in grayscale) at a base rate of 6 Hz (i.e., six images per second). At this rate, each stimulus lasts about 167 ms (i.e., 1 s/6). To minimize low-level repetition effects, face size randomly varied between 85 and 115% at every stimulus-onset (minimum step for size change: 5%). Each stimulation sequence presented the face of only one individual (Figure 1). The four individual faces were used and presented to the infants, in different stimulation sequences (i.e., with a different expression at the base). The association between an expression and an identity has been counterbalanced between infants. One target expression (i.e., disgust, happiness, or neutrality attributed to separate sequences) was presented at the base rate and a change of expression with the remaining five basic emotional expressions (except surprise) was introduced every 6th stimulus (i.e., at a lower rate of 6/6 = 1 Hz). For example, when neutrality is the target expression presented at the base rate, faces displayed at 1 Hz displayed in random order either happiness, disgust, anger, fear, or sadness. With this design, we test the discrimination from neutrality by opposing a neutral/unexpressive face (i.e., without facial actions) to all potential emotional facial expressions displayed randomly; this could isolate a marker of the detection of emotional/expressive facial configurations. By testing the discrimination from happiness, we also explore the discrimination of positive vs. negative/neutral (i.e., non-positive) emotions, all emotions but happiness being negative (4 out of 6) or unexpressive (1 out of 6) in our design. Consequently, this contrast may also capture the processing of emotional valence. Finally, when testing the discrimination from disgust, this negative expression is contrasted with expressions which are also negative (anger, fear, and sadness), but also positive (happiness) or neutral. Thus, this contrast tests the potential acquisition of a discrete status of disgust. In general, the approach dissociates two brain responses within a single stimulation sequence: the 6-Hz base frequency tags a general visual response to the rapid train of one individual face varying in size, while the 1-Hz expression-change rate tags a discrimination response selectively reflecting the perception of a change of expression from the target expression. All contrasted expressions were equally presented throughout each stimulation sequence, avoiding consecutive repetition. In sum, due to the periodic nature of the tagged EEG response that captures brain activities common to all expression changes within a sequence, the 1-Hz expression-change frequency indexes whether the infant brain discriminates five facial expressions from the target expression.

After EEG-cap placement, the infants were installed in a baby car seat in front of the screen in a dedicated light- and sound-attenuated Baby-lab. A camera placed on top of the screen continuously monitored them to check their well-being and attention to the screen. Each 34.5-s sequence started with a pre-stimulation interval of 0.5 s of blank screen, followed by a fade-in of increasing contrast for 1.833 s. Employed in earlier FPVS studies on infants (e.g., Leleu et al., 2020; Rekow et al., 2020, 2021), this sequence duration was thus well-adapted to the attentional span of infants and to technical issues (good signal to noise ratio; SNR). Full-contrast stimulation then lasted 31.167 s before a 0.833-s fade-out of decreasing contrast, and a post-stimulation interval of 0.167 s of blank screen. For each stimulation sequence, the target expression was displayed as the base rate and one of the five other expressions was randomly inserted every 6th image, at the 1-Hz rate of expression-change. Infants were constantly monitored *via* a webcam and stimulation sequences were launched when the signal was artifact-free and the infant was quietly looking at the screen. Auditory tones were transiently used to reorient infants’ attention toward the screen, without contaminating the frequency-tagged responses. Each infant was presented with the three target expressions (disgust, happiness, neutrality) in distinct sequences (Figure 1), their presentation order being counterbalanced across infants. Infants included in the final sample were exposed to 6–13 sequences, for a total testing duration ranging from 3.5 to 7 min.

### Electroencephalography recording and preprocessing

Electroencephalography was continuously recorded from 32 Ag/AgCl channels inserted in a cap (Waveguard, ANT Neuro, Netherlands) according to the 10-10 classification system. Channel AFz was used as reference during acquisition. Electrode impedance was kept below 40 kΩ and EEG was digitalized using ASAlab 4.7 (ANT Neuro, Netherlands) at a sampling rate of 1,024 Hz. EEG analyses were carried out using Letswave 6^1^ running on Matlab 2017 (MathWorks, United States). Left and right mastoid (M1 and M2) and prefrontal (Fp1, Fpz, Fp2) channels were removed before processing since they were noisy or artifact-ridden for most infants.

First, EEG data were bandpass filtered at 0.1–100 Hz using a 4th order Butterworth filter and resampled to 200 Hz. Data were then cropped for each sequence in segments lasting 36 s starting from the fade-in. To reduce high-amplitude artifacts, each segment was processed using the Artifact Blocking algorithm (Mourad et al., 2007; Fujioka et al., 2011) with a threshold of ±500 μV windowed on the overall segment (Leleu et al., 2020). For four 3.5-month-old infants a remaining noisy channel was rebuilt using linear interpolation from the nearest electrodes. As a result, only one channel was interpolated for four 3.5-month-olds (average: 0.22 ± 0.43 *SD*) and none for the 7-month-olds. Data were then re-referenced according to a common average reference and EEG data were further segmented in 32-s epochs from the start of the full-contrast sequence (i.e., 32 1-Hz cycles, removing the fade-in).

Two data-driven criteria were used for each infant to remove sequences when no general response was found to the fast train of the individual face changing in size, or when the 1-Hz expression-change response across the whole scalp presented with atypical noise-corrected amplitude compared to the other sequences [for a similar procedure, see Leleu et al. (2020) and Rekow et al. (2020, 2021)]. Fast Fourier transform (FFT) was first applied to every segment and amplitude spectra were extracted for all electrodes with a frequency resolution of 1/32 = 0.03125 Hz. The first criterion was based on Z-scores calculated for each channel and each frequency bin as the difference between the signal and the mean noise (estimation from the 20 surrounding bins, 10 on each side, excluding the two immediately adjacent and the two most extreme) divided by the standard deviation of the noise. According to previous FPVS-EEG studies showing a general response of the infant visual system to a 6-Hz stimulation sequence over medial occipital sites as a general marker of adequate looking at the stimulation screen (e.g., de Heering and Rossion, 2015; Peykarjou et al., 2017; Barry-Anwar et al., 2018; Leleu et al., 2020; Rekow et al., 2020, 2021), sequences were included in the analysis when at least two electrodes were associated with a Z-score above 1.64 (*p* < 0.05, one-tailed, signal > noise) or at least one electrode with a Z-score above 2.32 (*p* < 0.01, one-tailed) over medial occipital electrodes (Oz, POz, O1, O2) for the 6-Hz base frequency or the second harmonic (i.e., 12 Hz). For the second criterion, amplitude at each frequency bin was first corrected by subtracting the mean noise amplitude estimated from the six surrounding bins [for a similar procedure, see Leleu et al. (2020) and Rekow et al. (2020, 2021)]. Here, noise was estimated from fewer frequency bins since EEG amplitude is high in the low-frequency range with a non-linear decrease as frequency increases (Fransson et al., 2013). Hence, to consider too many bins would overestimate noise level (and therefore underestimate the expression-change response) because FFT amplitude spectrum is steeper for lower than for higher frequencies around the 1-Hz frequency. The global amplitude of the brain response over the scalp (i.e., square root of the sum of squared amplitudes of all channels) was then calculated at the 1-Hz expression-change frequency. A sequence was considered atypical when its global noise-corrected amplitude was above or below 2 *SDs* of the mean of all sequences (regardless of the expression) of the infant considered individually and retained after application of the first criterion. Once these two criteria were individually applied, between 6 and 14 sequences were kept per infant, with an average of 10.11 ± 1.78 *SD* for the 3.5-month-olds (for disgust condition: 3.44 ± 0.62 *SD*, range 2-4; for happiness: 3.22 ± 0.73 *SD*, range 2–4; for neutrality: 3.50 ± 0.71 *SD*, range 2–4), and an overall rejection of only 17 out of 199 sequences (8.54%). Similarly, in the 7-month-olds group, between 6 and 14 sequences were kept per infant, with an average of 10.56 ± 2.06 *SD* (for disgust condition: 3.56 ± 0.78 *SD*, range 2–5; for happiness: 3.56 ± 0.92 *SD*, range 2–6; for neutrality: 3.44 ± 0.86 *SD*, range 2–5), and an overall rejection of 24 out of 214 (11.2%). The resulting number of stimulation sequences was equivalent across conditions and age groups (i.e., 6–12 for 3.5-month-olds and 6–14 for 7-month-olds).

### Frequency-domain analysis

For each infant, the 32-s sequences were sorted according to each target expression condition and then averaged in the time-domain into a single epoch per condition. FFT was then applied to extract amplitude spectra for each electrode. To determine significant responses for both base and expression-change frequencies and their harmonics (i.e., integer multiples), amplitude at each channel was first normalized by dividing by the square root of the sum of squared amplitudes of all channels (McCarthy and Wood, 1985). Normalization was used to identify the main electrodes presenting a significant response by scaling differences between electrodes on the global power of the response across the scalp to determine whether the different expressions elicit distinguishable topographical patterns when scalp-wide amplitude is equalized across them. Then, data were grand-averaged across infants for each age group and Z-scores were calculated. For the general visual response at 6 Hz and harmonics, we considered electrodes located over the middle occipital cortex (Oz, POz, O1/2) as in previous FPVS-EEG infant studies (e.g., de Heering and Rossion, 2015; Peykarjou et al., 2017; Barry-Anwar et al., 2018; Leleu et al., 2020; Rekow et al., 2020, 2021). For the expression-change response, since this study is the first to investigate the response to facial expressions in infants with the FPVS-EEG approach, we first explored all electrodes over the scalp to identify those that showed a response for the different expressions at each age (*Z* > 1.96, *p* < 0.05, two-tailed). This bottom-up procedure allowed us to determine the electrodes that significantly responded to facial expressions [for a similar approach, see Dzhelyova et al. (2017) and Leleu et al. (2019)]. These electrodes were then included in the analyses if they were consistent with the electrodes that had been previously reported in other EEG studies of the brain response to facial expression in the first year (see below). Harmonics were considered for further analysis until Z-scores for two consecutive harmonics over one channel were no longer significant. For each response, individual normalized amplitudes were summed across significant harmonics (Retter et al., 2021) and corresponding Z-scores were calculated for these summed amplitudes for each infant and for grand-averaged data in each age group to estimate the significance of the overall responses at both group and individual levels. For illustration purpose, SNR of each response was computed on grand-averaged data as the amplitude (before normalization) divided by the mean amplitude of the noise (same estimation as for noise-corrected amplitudes, see above).

To analyze the differences between the three facial expression discrimination conditions and between age groups, each response was also quantified as a single value expressed in microvolts by summing noise-corrected amplitudes (before normalization) for significant harmonics. Individual summed noise-corrected amplitudes were extracted for each electrode with a significant response in at least one expression condition for at least one age group (as determined in the previous analysis; see Section “Results”). Based on the criteria exposed previously, for the expression-change response, we first explored electrodes located over occipito-temporal (Oz, O1/2, P7/8) and central regions (Cz, FC1/2, CP1/2) according to previous EEG studies on facial expression discrimination in infants (e.g., Nelson and de Haan, 1996; de Haan and Nelson, 1999; Halit et al., 2004; Leppänen et al., 2007; Xie et al., 2019). We selected electrodes O1/2, P7/8, T7/8, CP1/2, FC1/2 that are close to these locations and that showed a significant response in the previous analyses (see Section “Results”). For lateral electrodes, the homologous channel in the other hemisphere was also considered in the statistical analysis. A repeated-measures ANOVA was performed on the normalized noise-corrected amplitudes in both age groups using Expression (disgust, happiness, neutrality) and Electrode (POz, Oz, O1, O2 for the general visual response and O1/2, P7/8, T7/8, CP1/2, FC1/2 for the expression-change response) as within-subject factors. The factor Hemisphere (right, left) was also used as a within-subject factor for the expression-change response only. In addition, with the aim to directly explore the effect of age, we performed a repeated-measures ANOVA using Age (3.5- and 7-month-old) as a between-subject factor and Expression and Electrode as within-subject factors. For each analysis, Mauchly’s test for sphericity violation was performed and Greenhouse-Geisser correction was applied whenever sphericity was violated. Comparisons for significant effects were conducted using *T*-tests.

## Results

### Expression-change response

**FIGURE 2:**
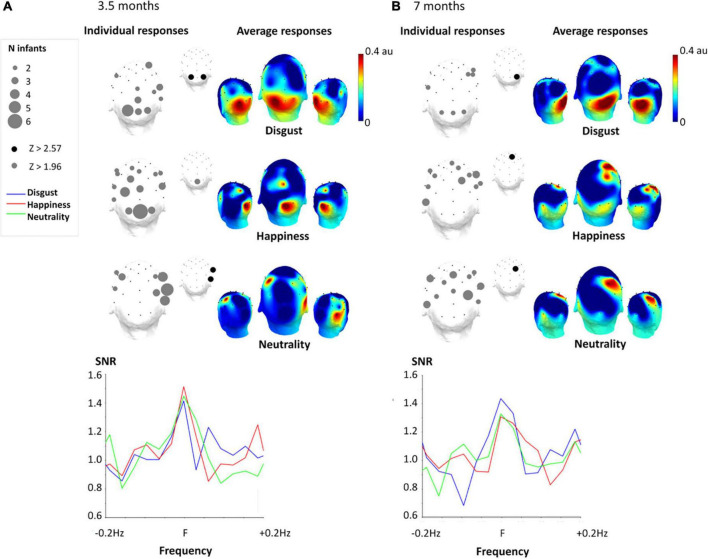
Expression-change response for the three facial expressions at 3.5 months **(A)** and 7 months **(B)**. Left part. Topographical representation (superior view) of significant individual responses. For each electrode, the number of infants with a significant response (*Z* > 1.96, *p* < 0.05) is represented by circle size when at least two individual responses were significant. The smaller topographical map also indicates significant electrodes at group level (gray: *Z* > 1.96, *p* < 0.05; black: *Z* > 2.57, *p* < 0.01). Right part. 3D topographical color maps (superior view) of the expression-change response (normalized noise-corrected amplitude in arbitrary units) for each expression condition. Bottom part. Signal-to-noise ratio (SNR) of the expression-change response and surrounding frequencies (±0.2 Hz, i.e., ±6 bins) averaged across significant electrodes for each expression condition.

In both age groups, exposition to rapid changes of expression (from either a disgust, happy, or neutral face) gave rise to identifiable brain responses with a high SNR (between 1.3 and 1.5; i.e., indicating 30–50% of signal increase compared with surrounding noise level), and with different scalp topographies (Figure 2; for topographies of non-normalized noise-corrected amplitudes, see Supplementary Figure 1). For 3.5-month-old infants (Figure 2A), the expression-change response for the target expression of disgust was significant for the 1st harmonic (i.e., 1 Hz) over the medial occipital channels O1 (*Z* = 2.97, *p* = 0.003) and O2 (*Z* = 4.34, *p* < 0.0001). For neutrality, the response was significant over the right occipito-temporal channel P8 until the 2nd harmonic (i.e., 2 Hz), with a significant response over T8 (*Z* = 2.93, *p* = 0.0034) and P8 (*Z* = 4.09, *p* < 0.001) when amplitude is summed across the two first harmonics. For happiness, only one electrode showed a significant response: Oz (*Z* = 2.05, *p* = 0.0404). At 7 months, every expression condition led to a significant expression-change response at the 1st harmonic. It was recorded over O2 (*Z* = 2.86, *p* = 0.0042) for disgust, over FC2 (*Z* = 2.79, *p* = 0.0053) for happiness and over CP2 (*Z* = 2.60, *p* = 0.0093) for neutrality (Figure 2B).

Altogether, these results reveal that, at 3.5 months of age, the brain response to a change of expression from disgust and happiness was concentrated over posterior regions, markedly over medial occipital sites. The response was more lateral for neutrality, with a right-hemispheric occipito-temporal distribution. In contrast, at 7 months of age, the expression-change response is still detected over the medial occipital region for disgust while recorded over central parietal sites for neutrality and over central frontal sites for happiness.

### Differences between expressions for each age group

To investigate the specific EEG response associated with the discrimination from the target expressions at each age, we first determined whether the distribution of the expression-change response over the scalp differentiates the different emotion categories for each age group. To do that, we analyzed normalized noise-corrected amplitudes (expressed in arbitrary units) over the different sites identified above and the corresponding site on the other hemisphere; namely, O1/2, P7/8, T7/8, CP1/2, and FC1/2. Medial electrodes were not included to investigate potential hemispheric differences.

**FIGURE 3:**
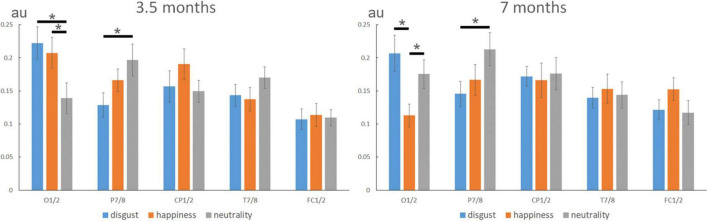
Expression-change response (normalized noise-corrected amplitudes, in arbitrary units) according to expressions over different sites (O1/2, P7/8, CP1/2, T7/8, and FC1/2) at 3.5 and 7 months. In 3.5-month-olds, the analysis revealed a similar topography for the two emotional expressions (i.e., disgust and happiness) that differed from neutrality with a lower contribution of O1/2 and a higher contribution of P7/8 for neutrality. In 7-month-olds, the topography of the expression-change response discriminated between disgust, neutrality, and happiness, with a lower contribution of O1/2 to the response to happiness compared to both disgust and neutrality. As for the 3.5-month-olds, P7/8 channels contributed more to the discrimination of neutrality than disgust. Note also the tendency for a higher contribution of FC1/2 to happiness, that was driven by a significant response over FC2 for happiness (see previous analyses). “*” indicated significant differences (p < 0.05).

In 3.5-month-olds, the analysis revealed a significant main effect of *Electrode* [*F*_(4,68)_ = 4.281, η*^2^_*p*_* = 0.201, *p* = 0.0038], with a larger expression-change response over O1/2 than over FC1/2, the other sites lying in between. More importantly for our purpose, the *Expression* × *Electrode* interaction was also significant [*F*_(8,136)_ = 2.886, η*^2^_*p*_* = 0.145, *p* = 0.0053; see Figure 3, left]. Complementary analyses using linear contrasts indicated that this interaction resulted from a different topography of the response to emotional expressions (i.e., disgust and happiness) compared to neutrality [*Expression* × *Electrode* interaction when disgust and happiness are pooled together: *F*_(4,68)_ = 6.244, *p* = 0.0002]. No significant difference emerged between disgust and happiness (*Expression* × *Electrode* interaction when neutrality is removed: *F* < 1). The difference between neutrality and the two emotional expressions resulted from a significantly lower contribution of O1/2 channels for the discrimination from neutrality [0.14 ± 0.02 (*SE*, standard error of the mean), arbitrary unit] than happiness (0.21 ± 0.02, *p* = 0.039), and disgust (0.22 ± 0.02, *p* = 0.004), together with a higher contribution of P7/8 electrodes for neutrality (0.20 ± 0.02) than disgust (0.13 ± 0.02, *p* = 0.029). No other main effect or interaction was significant for this age group.

In 7-month-olds, the only significant effect was the *Expression* × *Electrode* interaction [*F*_(8,136)_ = 2.210, η*^2^_*p*_* = 0.115, *p* = 0.0303; see Figure 3, right], and a trend for a larger response in the right hemisphere was noted [main effect of *Hemisphere*: *F*_(1,17)_ = 3.792, *p* = 0.0682]. Complementary analyses using linear contrasts indicated that by this age, and contrary to 3.5-month-old infants, the interaction was not explained by differences between emotional expressions and neutrality (*Expression* × *Electrode* interaction when disgust and happiness data are pooled: *F* < 1). Rather, the topography of the expression-change response was different between disgust and happiness [*Expression* × *Electrode* interaction when neutrality is removed: *F*_(4,68)_ = 3.42, *p* = 0.0122]. The interaction was mainly driven by a lower contribution of O1/2 to the response to happiness (0.11 ± 0.02) compared to both disgust (0.21 ± 0.03, *p* = 0.011) and neutrality (0.18 ± 0.02, *p* = 0.028). As for the 3.5-month-olds, P7/8 channels contributed more to the discrimination from neutrality (0.21 ± 0.02) than of disgust (0.15 ± 0.02, *p* = 0.019).

To directly assess the effect of age, we then performed a second ANOVA including *Age* (3.5- vs. 7-month-olds) as a between-subject factor. This analysis revealed a significant main effect of *Electrode* [*F*_(4,136)_ = 5.41, η*2p* = 0.134, *p* = 0.0004] and a significant *Expression* × *Electrode* interaction [*F*_(8,272)_ = 3.31, η*^2^_*p*_* = 0.089, *p* = 0.0013], but only a trend for the *Age* × *Expression* × *Electrode* interaction [*F*_(8,272)_ = 1.71, η*^2^_*p*_* = 0.048, *p* = 0.096].

### Individual expression-change responses

To assess the robustness of the expression-change EEG response at the individual level, we explored the responses across the whole scalp for each individual infant (see Supplementary Table).

Overall, individual responses confirm group-level observations with the discrimination of facial expression from disgust mainly eliciting medial occipital expression-change responses at both ages despite a broad distribution over the scalp (Figure 2). In contrast, while expression changes from happiness and neutrality lead to posterior brain responses for the 3.5-month-olds (i.e., over the medial occipital and right lateral channels respectively), they give rise to more anterior activity for happiness in 7-month-old infants. In sum, the expression-change response is reliably found at the individual level ensuring that the group response is not accounted for by a small subset of infants.

### General visual response

**FIGURE 4:**
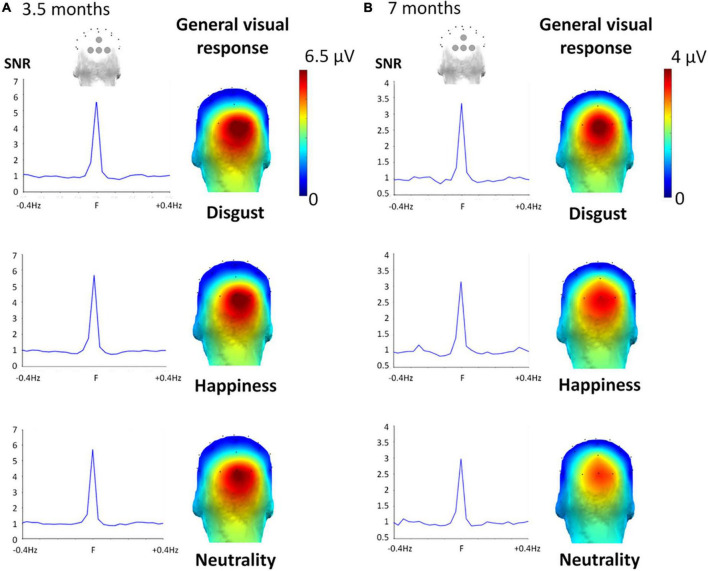
General visual response for the three target facial expressions at 3.5 months **(A)** and 7 months **(B)**. Left part. SNR was calculated on the summed harmonics values averaged between the four occipital electrodes, considering Z scores of at least one of the four occipital electrodes was significant (across harmonics *Zs* > 2.60, *p* < 0.006), showing a high SNR for each expression: around six for every expression-condition in the 3.5-month-olds, and around three in the 7-month-olds. Right part. Topographical 3D map (back view) shows a clear medial occipital cortex response of the noise-corrected amplitude (in μV) values summed until the 6th harmonic of the 6 Hz response for the three conditions.

As expected, the 6-Hz stimulation elicited a clear brain response at the same frequency and its harmonics (e.g., 12, 18 Hz) over the medial occipital cortex, reflecting the general visual processing of the rapidly presented individual faces changing in size (Figure 4). Exploration of the significant electrodes for each facial expression condition and each age group revealed a significant response at 6 Hz over the four occipital channels (Oz, POz, O1, O2) for every expression condition and both age groups (all *Zs* > 3.06, *ps* < 0.0022). Following harmonics were significant over at least one of these electrodes until the sixth harmonic (i.e., 36 Hz) for every expression and age group (all *Zs* > 2.59, *ps* < 0.0096). When summed across harmonics, the general visual response was still significant over the 4 medial occipital channels (all *Zs* > 7.46, *ps* < 0.0001). SNR was very high in 3.5-month-olds (SNR ≈ 6, i.e., signal six times larger than noise) and lower but still high in 7-month-olds (SNR ≈ 3, i.e., signal three times larger than noise) (Figure 4). Importantly for our purpose, the repeated-measures ANOVAs run separately at each age revealed that no effect involving the factor *Expression* was significant.

The second ANOVA revealed a trend for the effect of *Age* [*F*_(1,34)_ = 3.69, η*^2^_*p*_* = 0.098, *p* = 0.063], with a larger response in the 3.5-month-olds [5.22 ± 0.95 μV (SE)] than in the 7-month-olds (3.21 ± 0.44 μV). In addition, we found a significant main effect of *Electrode* [*F*_(1.93,65.64)_ = 15.83, η*^2^_*p*_* = 0.64, *p* < 0.0001] qualified by a significant *Electrode* × *Age* interaction [*F*_(1.93,65.64)_ = 5.39, η*^2^_*p*_* = 0.64, *p* = 0.007]. This latter interaction was characterized by a significant difference between age groups (3.5 vs. 7-month-olds) for electrodes O2 (6.32 ± 1.19 μV vs. 3.42 ± 0.43 μV, *p* = 0.03), Oz (6.91 ± 1.31 μV, vs. 3.83 ± 0.65 μV, *p* = 0.04), together with a trend for O1 (4.84 ± 0.94 μV, vs. 2.90 ± 0.46 μV, *p* = 0.07) and no difference for POz (2.84 ± 0.54 μV, vs. 2.69 ± 0.53 μV, *p* = 0.85).

## Discussion

Using FPVS-EEG, the present study isolated direct brain markers of rapid facial expression discrimination in 3.5- and 7-month-old infants, by investigating the specific neural responses to brief changes of facial expression within rapid streams of neutral, disgust, or happy faces. A significant expression-change response was observed over posterior scalp regions for the discrimination from disgust and from happiness in 3.5-month-olds, with a similar response for the discrimination from disgust in 7-month-olds. For the discrimination from neutrality, the expression-change response was recorded over more anterior, occipito-temporal and parietal regions, in both age groups. Finally, a response to a change of expression from happiness emerged over central frontal scalp regions at 7 months of age. These results show that both 3.5- and 7-month-olds discriminate several facial expressions from each target expression. They also suggest that distinct brain regions/neural networks could be involved in facial expression discrimination depending on the age of the infants and the expression.

For the discrimination from neutrality, by opposing a neutral/unexpressive face (i.e., without facial actions) to all potential emotional facial expressions displayed randomly, we isolated a brain response to the occurrence of facial actions within the face at both ages. Thus, this response could be a marker of the detection of expressive facial configurations. In infants, ERP studies revealed several cortical sources within occipital brain areas (notably the IOG), the latFG, the posterior temporal cortex (including the pSTS), the PCC/precuneus or the middle frontal cortex (including the medial prefrontal cortex: mPFC) (Guy et al., 2016; Xie et al., 2019), suggesting that the neural network delineated in adults is already partly functional during the first year of life (Leppänen and Nelson, 2009; Leppänen, 2011). Given that the expression-change response for neutrality was recorded over scalp regions analogous to those observed in adults [i.e., at right occipito-temporal sites; e.g., Poncet et al. (2019)], our findings might suggest that this network is already functional at 3.5 months of age, and subtends the discrimination of neutral vs. expressive faces. However, considering the lack of evidence for more finely tuned processes that distinguish the different expression categories at this age (Campos and Stenberg, 1981; Oster, 1981; Walker-Andrews, 1997; Leppänen and Nelson, 2009; Quinn et al., 2011), the functionality of this network could be limited to segregate any expressive face from a neutral face irrespective of the emotional content. It worth noting that these interpretations remain tentative as the analogy between topographies in infants and adults must be made with caution, and scalp EEG is limited by its coarse spatial resolution. In addition, we cannot ascertain that the response is specific to *emotional* configurations. Further research is needed to dismiss the possibility that opposing neutral faces to facial expressions without emotional content, such as tongue protrusion or speech-related facial movements (e.g., say “O”), would elicit a similar neural response.

An expression-change response from disgust and happiness was also isolated at both ages. This response was recorded over medial occipital scalp regions in 3.5-month-olds, and over medial occipital and fronto-central regions, for disgust and happiness respectively, in 7-month-olds, both regions responding more than to the expression-change from facial neutrality. This observation suggests that the infant brain discriminates several expressions from every target expression at both ages. Indeed, the expression-change response emerges only if the brain detects visual cues that both (1) occur reliably in the target expression, and (2) do not systematically occur in the other expressions. These cues can be either local properties of a single facial action (e.g., a smiling mouth or a wrinkled nose), or more complex and integrated patterns (i.e., the co-occurrence of facial actions over the whole face). In particular here, for the brain response to a brief change of expression from neutrality, the changes were associated with facial actions turning from unexpressive (i.e., neutral face) to expressive (i.e., anger, disgust, happiness, fear, or sadness) over the whole face. In contrast, the brain responses to disgust and happiness were not driven by all facial features at each change since facial actions can be shared across expressions (e.g., eyebrow lowering for anger, disgust, fear, and sadness). One may therefore suggest that discriminating from neutrality relies more on brain structures that integrate the configuration of facial actions over the whole face, whereas discriminating from both disgust and happiness is subtended by lower-level regions that process more local information. In sum, we propose that the medial occipital response to an expression change from disgust and happiness in 3.5-month-olds, and from disgust in 7-month-olds, could be related to the discrimination of local facial actions that differentiate several expressions from the target expression (e.g., the smiling mouth in happy faces, the wrinkled nose in disgust faces). By contrast, the more lateral response observed for an expression-change from neutrality could be elicited by the co-occurrence of several expressive features over the whole face, regardless of the (emotional) nature of the configuration of facial actions.

Contrary to the discrimination from disgust and neutrality, the brain response to a change of expression from happiness differed between 3.5 and 7 months. The response mainly appeared over occipital/posterior sites and was not different from the response to the discrimination from disgust in 3.5-month-olds, whereas the occipital response contributed less to the expression-change from happiness than other categories in 7-month-olds. Rather, a change of expression from happiness elicited a response over the central frontal region at this age. This topographic shift might reflect a specialization of the brain response, with the integration of affective and/or social meaning, as demonstrated by recent studies (e.g., Palama et al., 2018). The scalp topography observed here is consistent with those reported in ERP studies (notably with the topography of the “Negative Central”: Nc component) in situations supposed to involve different levels of interest for infants [e.g., new vs. familiar objects, Reynolds and Richards (2005); faces vs. toys, Guy et al. (2016)], or for different expressions (e.g., Xie et al., 2019). These findings were thought of as reflecting stimulus salience (Nelson and de Haan, 1996) or attentional processes (e.g., Richards, 2003; Reynolds and Richards, 2005; Guy et al., 2016; Xie and Richards, 2016; Xie et al., 2019). For example, Xie et al. (2019) proposed that a central frontal response emerges for expressions that engage attention allocation and deeper processing. Similar central frontal activities were reported in response to happiness using fNIRS (Minagawa-Kawai et al., 2008; Fox et al., 2013). These findings were explained by the acquisition of socio-cognitive abilities (Grossmann, 2013).

An alternative (although non-exclusive) hypothesis is that the brain response to a rapid change of expression from happiness indexes the discrimination of positive vs. negative emotions at 7 months. In our design, all emotions but happiness were negative (4 out of 6) or unexpressive (1 out of 6). Thus, the brain response isolated by this contrast may also capture the processing of emotional valence, and not solely the processing of visually distinct expressions. However, irrespective of the nature of the response, it indicates that discriminating several expressions from happiness elicits a different brain response in 7-month-olds, possibly reflecting more attention or the recruitment of specific brain mechanisms implicated in affective, cognitive and/or social processing. Future studies should examine the hypothetical relationship between central frontal activities and emotional meaning attribution, for instance by testing the influence of contextual information, such as the emotional environment provided by the mother (de Haan et al., 2004; Jessen, 2020) or the multisensory context provided by auditory (Flom and Bahrick, 2007) or odor cues (Godard et al., 2016).

The brain response to an expression change from disgust was mainly found over occipital/posterior sites and did not evolve with age. It indicates that the brain has detected some visual cues that reliably occur in a disgust face and are absent in the other expressions (i.e., anger, happiness, fear, sadness, and neutrality). Considering the facial actions identified for these different expressions, the main candidates for disgust-specificity are nose wrinkling and lips parting together with upper lip rising; two actions more associated with disgust than with any other facial emotions (Ekman et al., 1978). The stability of the response between 3.5 and 7 months suggests that this expression is similarly processed during this period, contrary to happiness. This suggestion is in line with behavioral studies, which indicate that the emotional meaning of disgust faces is not integrated before 12 months (Moses et al., 2001; Hertenstein and Campos, 2004) or even later (Widen and Russell, 2010, 2013), as its understanding would imply a higher cognitive development (Rozin and Fallon, 1987; Widen and Russell, 2013). It may also be possible, however, that the contrast performed here did not allow to isolate the specific response to the emotional meaning of this facial expression. As mentioned earlier, the contrasted expressions were mainly negative (four expressions: disgust, anger, fear, and sadness), as opposed to only one positive expression (happiness) and neutrality. Thus, we cannot exclude that a face expressing disgust already acquired the status of a negative signal at 7 months, but may not be dissociated from other negative facial signals, except from visual characteristics (as suggested by the occipital expression-change response). This hypothesis should be further investigated by testing other negative emotions, such as fear or anger, already known to trigger infants’ attention in relation to meaning attribution (Leppänen and Nelson, 2009; Xie et al., 2019).

Finally, as a limitation, it is worth noting that the different patterns of brain activity observed for each discrimination at each age were only partially supported by a trend for a 2-way interaction in the global analysis that included age as a factor. At least two main reasons can explain this finding. First, contrary to the response to the discrimination from happiness, the responses to the discrimination from disgust and neutrality appear similar at each age. The analysis including the three conditions was thus probably limited in its ability to evidence an effect of age that is entirely driven by only one condition. In addition, several confounding factors (e.g., brain maturation, skull thickness) may also lead to differences in the amplitude and topography of the brain responses between the two age groups, and partially hinder our ability to identify the effect of age on the response to a discrimination from happiness. Future studies should thus further investigate the brain responses to different facial expressions at different ages using complementary approaches.

## Conclusion

Using FPVS-EEG, we characterized brain responses indicating that several basic facial expressions are discriminated from the expressions of disgust, happiness and neutrality at 3.5 and 7 months of age. The response to a change of expression from disgust was mainly located over medial occipital sites at both ages, likely reflecting visual discrimination based on local facial features. The distinct response noted at both ages for the expression-change from neutrality further suggests that the discrimination from this expression relies on more global cues (i.e., integration of facial actions over the whole face). Finally, for the discrimination from happiness, the expression-change response was recorded over the occipital region at 3.5 months, while we rather found a significant response over central frontal scalp regions at 7 months, potentially reflecting a critical developmental change in the processing of the emotional content of smiling faces.

## Data availability statement

The original contributions presented in this study are included in the article/Supplementary material, further inquiries can be directed to the corresponding authors.

## Ethics statement

The studies involving human participants were reviewed and approved by Comité de Protection des Personnes Sud-Est III–2016-A02056-45. Written informed consent to participate in this study was provided by the participants’ legal guardian/next of kin. Written informed consent was obtained from the individual(s) for the publication of any potentially identifiable images or data included in this article.

## Author contributions

FP, AL, DR, and J-YB contributed to the design and implementation of the research, to the analysis of the results, and to the writing of the manuscript. FD contributed to the implementation of the research and to the writing of the manuscript. MD contributed to the design of the research and to the writing of the manuscript. BS, KD, and LF contributed to the writing of the manuscript. BR contributed to the design of the research, to the analysis of the results, and to the writing of the manuscript. DR contributed to the design and implementation of the research and to the writing of the manuscript. All authors contributed to the article and approved the submitted version.

## Footnotes

1
http://nocions.github.io/letswave6
