# Supplementary material for: A neural marker of rapid discrimination of facial expression in 3.5- and 7-month-old infants
Source: Front Neurosci. 2022 Aug 18;16:901013. doi: 10.3389/fnins.2022.901013 (PMC9434348; doi:10.3389/fnins.2022.901013)
Supplement: Supplementary file 1 [file Data_Sheet_1.docx]

**Supplementary material - A neural marker of rapid discrimination of facial expression in 3.5- and 7-month-old infants**

**
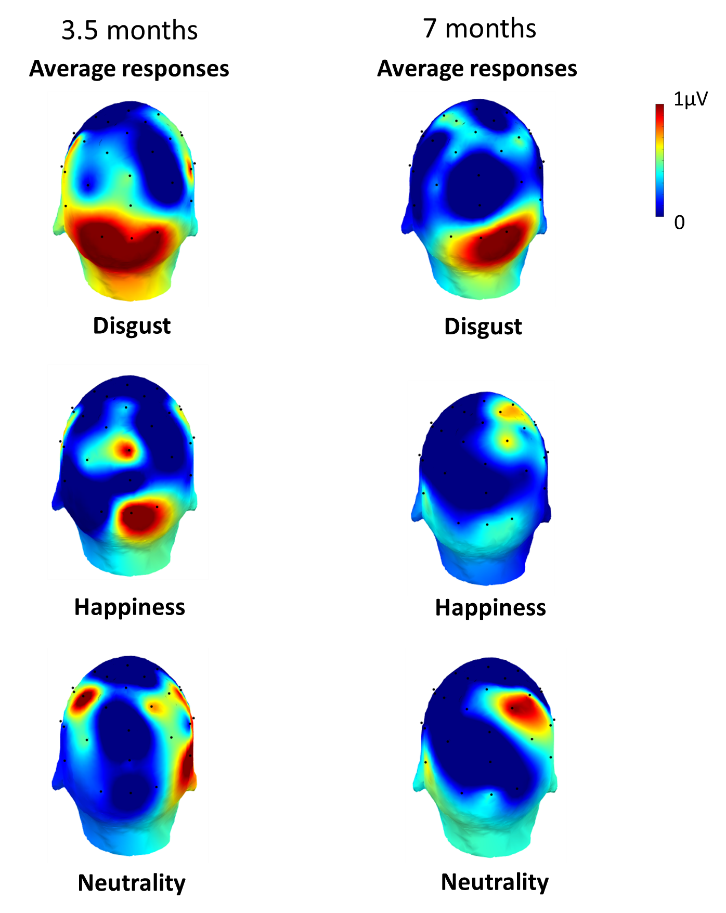
**

*Supplementary figure S1.* **3D-topographical maps (superior view) of the expression-change response (noise-corrected amplitudes) for each facial expression and age group.**

Supplementary tables

*Electrodes with a significant response (Z > 1.96, p< .05, two-tailed) and the associated p value for each infant and each expression for the 3.5 and the 7-month-old groups, respectively.*

| **3.5-month-olds** | **Disgust** | **Happiness** | **Neutrality** |
| --- | --- | --- | --- |
| **INF1** | CP1 ^p=.037^, O1 ^p=.002^, Oz ^p=.049^, POz ^p=.001^ | C4 ^p=.019^, F8 ^p=.030^, FC1 ^p=.020^, O1 ^p=.013^, Oz ^p=.021^ | None |
| **INF2** | O1 ^p=.012^ | FC5 ^p=.047^ | F4 ^p=.020^, FC5 ^p=.040^, P4 ^p=.005^ |
| **INF3** | P7 ^p=.003^ | CP5 ^p=.007^, P7 ^p=.005^ | P8 ^p=.005^, T8 ^p=.035^ |
| **INF4** | O2 ^p=.023^, POz ^p=.024^, Pz ^p=.013^ | O1 ^p=.021^, O2 ^p=.016^, Oz ^p=.022^, P8 ^p=.043^, POz ^p=.039^ | P4 ^p=.039^ |
| **INF5** | F4 ^p=.003^ | C3 ^p=.050^, FC2 ^p=.016^, P3 ^p=.011^, Pz ^p=.035^ | P8 ^p=.041^, POz ^p=.001^ |
| **INF6** | CP6 ^p=.001^ | FC6 ^p=.003^ | C3 ^p<.001^, F7 ^p=.037^ |
| **INF7** | F4 ^p<.001^, FC5 ^p<.001^ | CP1 ^p=.009^, O1 ^p=.003^, Pz ^p=.005^ | C3 ^p=.014^, CP6 ^p=.001^, P4 ^p=.007^, P8 ^p=.047^, T8 ^p=.026^ |
| **INF8** | O1 ^p=.002^, P4 ^p=.015^ | CP6 ^p=.047^, Cz ^p<.001^, P4 ^p=.027^, T7 ^p=.003^ | FC6 ^p=.027^ |
| **INF9** | P8 ^p=.039^, POz ^p=.003^ | Oz ^p=.044^, Pz ^p<.001^ | F7 ^p=.014^ |
| **INF10** | CP5 ^p=.030^, CP6 ^p=.002^, O1 ^p=.013^ | F7 ^p=.022^, P3 ^p=.018^ | CP5 ^p=.004^, T8 ^p=.001^ |
| **INF11** | P8 ^p=.035^, T8 ^p=.017^ | CP5 ^p=.004^, Cz ^p=.009^, FC6 ^p=.047^, O2 ^p<.001^, Oz ^p=.001^, T8 ^p=.019^ | C4 ^p=.042^ |
| **INF12** | F4 ^p=.019^, FC2 ^p=.016^, O2 ^p<.001^, Oz^p<.001^ | None | FC6 ^p=.044^ |
| **INF13** | None | FC5 ^p=.004^, P3 ^p=.020^ | CP1 ^p<.001^, FC6 ^p=.005^ |
| **INF14** | Cz ^p=.011^, Oz ^p=.004^ | C4 ^p=.022^, Oz ^p=.039^, P3 ^p=.005^ | C3 ^p=.042^, CP5 ^p<.001^, P8 ^p=.042^, T8 ^p=.038^ |
| **INF15** | CP6 ^p=.040^, P4 ^p=.022^ | CP1 ^p=.004^, CP6 ^p=.011^, P7 ^p=.001^ | P7 ^p=.007^ |
| **INF16** | CP2 ^p=.002^, FC5 ^p=.035^ | CP1 ^p=.001^, CP6 ^p=.011^, F3 ^p=.017^, F7 ^p=.030^, FC1 ^p<.001^, Fz ^p=.019^ | C4 ^p=.003^, FC6 ^p=.026^ |
| **INF17** | O2 ^p=.006^ | None | T8 ^p=.037^ |
| **INF18** | O1 ^p<.001^, Pz ^p=.038^ | O2 ^p<.001^, Oz ^p=.004^ | None |

| **7-month-olds** | **Disgust** | **Happiness** | **Neutrality** |
| --- | --- | --- | --- |
| **INF1** | O2 ^p=.021^, Oz ^p=.001^, T7 ^p=.049^ | POz ^p=.034^ | F8 ^p=.017^, FC5 ^p=.013^ |
| **INF2** | CP5 ^p=.013^ | None | C4 ^p=.023^ |
| **INF3** | FC2 ^p=.007^, FC6 ^p=.001^, Fz ^p=.001^ | CP5 ^p=.013^, FC5 ^p=.004^, O1 ^p=.031^ | FC5 ^p=.009^, P7 ^p=.038^ |
| **INF4** | none | C3 ^p=.011^, FC2 ^p=.037^ | Oz ^p=.009^, P3 ^p=.003^, P4 ^p=.018^ |
| **INF5** | CP6 ^p=.031^, O2 ^p=.020^ | C4 ^p=.048^, Fz ^p<.001^, P8 ^p=.025^ | CP2 ^p<.001^ |
| **INF6** | O2 ^p<.001^ | CP2 ^p=.013^ | CP6 ^p=.029^, FC1 ^p<.001^, FC5 ^p<.001^ |
| **INF7** | C4 ^p=.009^, F4 ^p=.038^ | CP6 ^p=.023^, Pz ^p<.001^ | C4 ^p=.018^, P7 ^p=.006^ |
| **INF8** | None | None | CP2 ^p=.045^, P3 ^p=.049^, Pz ^p<.001^, T8 ^p=.003^ |
| **INF9** | FC6 ^p=.002^ | C4 ^p=.002^, FC6 ^p=.035^ | P8 ^p<.001^ |
| **INF10** | CP5 ^p=.023^, FC1 ^p=.009^, O1 ^p=.002^ | C3 ^p=.026^, F7 ^p=.023^, POz ^p=.038^ | CP5 ^p=.020^ |
| **INF11** | C3 ^p=.019^ | CP2 ^p=.033^, CP6 ^p<.001^, FC6 ^p<.001^ | P7 ^p=.036^ |
| **INF12** | CP1 ^p=.003^, O1 ^p=.019^ | FC6 ^p=.018^, T8 ^p=.003^ | None |
| **INF13** | CP2 ^p=.002^, F8 ^p=.028^ | None | CP1 ^p=.041^, CP2 ^p=.048^, Cz ^p=.001^ |
| **INF14** | C4 ^p=.004^ | F8 ^p=.005^, P7 ^p=.005^ | Pz ^p=.019^, T8 ^p=.033^ |
| **INF15** | Cz ^p=.002^, P3 ^p=.012^ | CP2 ^p<.001^, CP5 ^p=.019^, P7 ^p=.048^, T8 ^p=.044^ | CP1 ^p=.006^, Cz ^p=.003^ |
| **INF16** | F8 ^p=.032^ | P7 ^p=.002^ | CP2 ^p=.034^ |
| **INF17** | Oz ^p=.017^ | F4 ^p=.018^, Oz ^p=.001^ | T8 ^p<.001^ |
| **INF18** | F3 ^p=.009^ | Cz ^p=.030^, F7 ^p=.002^, FC2 ^p=.004^, Fz ^p=.009^ | Cz ^p=.014^, F8 ^p=.003^, P8 ^p=.004^ |
